# 

**Published:** 1879-07

**Authors:** 


### ARTICLE IX.

### DENTAL ASSOCIATIONS.

### *Virginia State Dental Association.*

At the last annual meeting of the Virginia Dental Association it was determined to make a "new departure," changing the time and place of holding the annual meeting, hoping thereby to secure a larger attendance and more enthusiasm than has marked our meetings for several years past.

In accordance with this determination, the regular annual meeting for this year will be held in the city of Charlottesville, Tuesday, August 19th, at 10 o'clock A. M.

It is hoped that every member of the Association will use his best endeavors to be present, and lend his aid in making the meeting interesting and profitable.

All members of the profession, not only in Virginia, but in sister States, whether members of the Society or not, are cordially invited to meet with us and take part in our discussions.

I most earnestly request that each member of the several standing committees will prepare an individual report, and not depend upon the other members of the committee to do the duty for him. This it will be seen the Constitution of the Society requires; and it is only by faithfulness in this matter that we can expect to accomplish the objects of our meeting. It is hoped that those who find themselves unable to attend in person, will forward their papers to Dr. W. E. Norris, Charlottesville, in time to be read at the meeting.

Past experience has shown that when the committees are announced early in the year, the members procrastinate and forget the appointment. The issuing of this notice has been delayed, in the hope that those appointed on the committees would see the necessity for setting to work at once, to have their reports ready at the time of meeting.

L. M. Cowardin, *Corresponding Secretary.*

J. Hall Moore, *President.*

---

### *National Dental Association.*

The Eleventh Annual Meeting of this Association, (late Southern,") will be held in Augusta, Georgia, commencing on the 2nd Tuesday in July, 1879, at 10 A. M.

It is expected that the members of the Georgia, South Carolina and North Carolina State Dental Societies will be present, and a cordial invitation is extended to the profession generally.

OFFICERS.

*President.*—Prof. F. J. S. Gorgas, Baltimore, Md.

*1st Vice President.*—Dr. L. D. Carpenter, Atlanta, Ga.

*2nd Vice President.*—Dr. J. R. Walker, New Orleans, La.

*3rd Vice President.*—Dr. John G. Wayt, Richmond, Va.

*Cor. Secretary.*—Dr. A. C. Ford, Atlanta, Ga.

*Rec. Secretary.*—Dr. E. S. Chisholm, Tuscaloosa, Ala.

*Treasurer.*—Dr. H. A. Lowrance, Athens, Ga.

*Executive Committee.*—Drs. W. C. Wardlaw, G. H. Winkler, of Augusta; G. W. H. Whitaker, Sandersville, Ga.

The members of the different Standing Committees are earnestly requested to be present, and present papers on the subjects designated. Proper arrangements will be made for reduction of Rail Road fares, &c., by the Executive Committee.

E. S. CHISHOLM,
*Recording Secretary.*

---

*South Carolina State Dental Association.*

The Ninth Annual Meeting of the South Carolina State Dental Association will be held in the office of the President, J. B. Patrick, D. D. S., July 7th, 1879, at 10 A. M., in Charleston, S. C. That afternoon the Association will adjourn to meet in joint session with the Southern Dental Association and Georgia Dental Society, next morning, July 8th, at Augusta, Ga.

All respectable dentists are informed, that it is their privilege and duty to attend one or both of these meetings. Every inducement which can, will be afforded the profession.

The State Board of Dental Examiners will meet at Charleston, in the office of Dr. J. B. Patrick, July 7th, 1879, at 10 A. M.

Candidates for the practice of dentistry in South Carolina must apply, at or before that time, to Dr. W. S. Brown, of Charleston.

The attention of graduates in dentistry subsequent to February 23, 1875, is called to Sections 3rd and 7th of an Act of the General Assembly, to regulate the practice of dentistry in this State. G. F. S. Wright.

*Rec. Secretary, Columbia S. C.*

---

### *American Dental Association.*

The Local Committee of the American Dental Association report by their Chairman, Dr. G. L. Field, the following provisions for the Nineteenth Annual Session of this Association, to be held at Niagara Falls, August 5th, 6th, 7th, and 8th, 1879.

The Pavilion in Prospect Park has been engaged for the Meetings. Each person attending will be charged one entrance, (25 cents,) to the Park for the Session.

Hotel charges reduced to $3,00 per day at the Cataract House and International Hotel. Half rates to all places of interest in the vicinity. One ticket good for the Session. Certificates entitling members to reduced fares over the Great Western and Wabash Rail Roads may be obtained of Dr. George L. Field, Detroit, Michigan, previous to August 1st. Reduced rates may be had over the Erie and New York Central Rail Roads after July 15th, of Dr. F. M. Odell, No. 7 West 38th Street, New York.

Tickets will be issued over the Cincinnati Hamilton and Dayton and Canada Southern Rail Roads, from Cincinnati to Niagara Falls and return for $10,00.

Thomas Fillebrown,

*Ch'n 1st Div. Ex. Committee.*

---

### *Georgia State Dental Society.*

The Eleventh Annual Session of the Georgia State Dental Society will be held in the city of Augusta, Ga., on Tuesday, the 8th of July next, at 10 o'clock A. M.

The State Board of Dental examiners meets at the same time and place.

L. D. Carpenter,

*Cor. Secretary.*

*The American Dental Convention*

Will hold its 25th Annual Session in Saratoga on the Second Tuesday, (12th) of August, 1879. The Committee have made arrangements by which members will be entertained at reduced rates at the United States Hotel.

Provision has been made for the exhibition of Dental Materials, Appliances, &c. Manufacturers and Venders are invited to exhibit.

The profession generally are earnestly invited to attend this Session, which is expected to be of more than usual interest.

All reputable practitioners are eligible to membership.

For further information address J. G. Ambler, 25 West 23rd Street, New York.

J. G. Ambler,
John Allen,
L. S. Struw,
C. F. Rich,
*Committee of Arrangements.*

### To Readers and Correspondents.

Communications intended for publication, and exchange journals and papers, should be addressed to the Editor of the American Journal of Dental Science, 259 N. Eutaw St., Baltimore, Md. All letters relating to the business of the journal, or containing cash remittances, to be directed to Messrs. Snowden & Cowman. Articles for publication should be received by the editor at least three weeks previous to the first day of the month on which the number in which it is designed for them to appear, is issued.

Article IX.—Dental Associations—Virginia State Dental Association ........ 131

Nationa. Dental Association ........ 132

South Carolina State Dental Association ........ 133

American Dental Association ........ 134

Georgia State Dental Society ........ 134

American Dental Convention ........ 135

### EDITORIAL, ETC.

Indiana Medical Protective Law ........ 136
Loss & Gain ........ 137
Storms & Neuralgia ........ 139
Orkney Springs, Virginia ........ 140

### MONTHLY SUMMARY.

☞ The American Journal of Dental Science will contain fifty pages of reading matter in each number, making a volume of about

*Six Hundred* pages

EXCLUSIVE OF ADVERTISEMENTS

THE

### AMERICAN JOURNAL
OF
### DENTAL SCIENCE.

The *Journal* is issued on the first of each month and contains not less than *fifty* pages of reading matter in each number, exclusive of advertisements, making a volume of *six hundred* pages per annum.

In each number will be found Original Communications, Correspondence, Selected Articles, Monthly Summary, Bibliographical Notices and Editorial Matter, and every effort will be exerted to render the *Journal* worthy of the patronage of the Dental profession.

A generous co-operation is respectfully solicited from the members of the profession; and as the *Journal* has now a printing office of its own, which materially lessens the cost of its publication, it will be issued at the reduced subscription price of

**$2.50 Per Annum in Advance.**

Communications are respectfully solicited on all subjects pertaining to the practice of dentistry, as well as reports of cases occurring in dental practice.

### RATES OF ADVERTISING.

| | 1 mo. | 2 mo. | 3 mo. | 6 mo. | 12 mo. |
|---|---|---|---|---|---|
| 1 Page. | $15 00 | $22 50 | $30 00 | $50 00 | $100 00 |
| ½ " | 10 00 | 15 00 | 20 00 | 30 00 | 50 00 |
| ¼ " | 7 00 | 11 00 | 15 00 | 20 00 | 30 00 |
| ⅛ " | 5 00 | 8 00 | 11 00 | 15 00 | 20 00 |

All original communications designed for publication, works for review, new instruments for notice, exchanges, &c., should be directed to the editor, 259 N. Eutaw St., Baltimore, Md.

All advertisements and subscriptions should be addressed to

SNOWDEN & COWMAN,
*Publishers of the Am. Journal of Dental Science.*
86 W. Fayette St. Bet. Charles & Liberty Sts.,
BALTIMORE, MD.

